# Challenges of Iranian Clinicians in Dealing with COVID-19: Taking Advantages of The Experiences in Wenzhou

**DOI:** 10.22074/cellj.2020.7604

**Published:** 2020-09-08

**Authors:** Yuping Li, Yaser Tahamtani, Mehdi Totonchi, Chengshui Chen, Seyed Mohammad Reza Hashemian, Fatemeh Amoozegar, Jin-San Zhang, Yousef Gholampour, Xiaokun Li

**Affiliations:** 1. Department of Pulmonary and Critical Care Medicine, The First Affiliated Hospital of Wenzhou Medical University, China; 2. Department of Diabetes, Obesity and Metabolism, Cell Science Research Center, Royan Institute for Stem Cell Biology and Technology, ACECR, Tehran, Iran; 3. Department of Stem Cells and Developmental Biology, Cell Science Research Center, Royan Institute for Stem Cell Biology and Technology, ACECR, Tehran, Iran; 4. Department of Genetics, Reproductive Biomedicine Research Center, Royan Institute for Reproductive Biomedicine, ACECR, Tehran, Iran; 5. Chronic Respiratory Diseases Research Center, National Research Institute of Tuberculosis and Lung Diseases (NRITLD), Shahid Beheshti University of Medical Sciences, Tehran, Iran; 6. Noncommunicable diseases research center, Fasa University of Medical Sciences, Fasa, Iran; 7. International Collaborative Center on Growth Factor Research, and School of Pharmaceutical Sciences, Wenzhou Medical University, China; 8. Department of Internal Medicine, School of Medicine, Fasa University of Medical Sciences, Fasa, Iran; 9. School of Pharmaceutical Sciences, Wenzhou Medical University, and Wenzhou Biomedicine Collaborative Innovation Center, Wenzhou, China

**Keywords:** Anti-inflammatory Drug, Antiviral Drug, COVID-19, Diagnosis, SARS-CoV-2

## Abstract

The novel coronavirus has been spreading since December 2019. It was initially reported in Wuhan, Hubei province of China. Coronavirus disease 2019 (COVID-19) has currently become a pandemic affecting over seven million people worldwide, and the number is still rising. Wenzhou, as the first hit city out of Hubei Province, achieved a remarkable success in effectively containing the disease. A great record was also observed in Wenzhou for the clinical management of COVID-19 patients, leading to one of the lowest death rates in China. Researchers and clinical specialists proposed and formulated combined approaches such as computerized tomography (CT)- scans and molecular assays, as well as using both allopathic and traditional medications to mitigate its effects. Iranian and Chinese specialists and scientists had a communication in clinical, molecular and pharmaceutical aspects of COVID-19. A proper guideline was prepared according to the experiences of Chinese clinicians in managing the full spectrum of COVID-19 patients, from relatively mild to highly complex cases. The purpose of this guideline is to serve a reference in the hospital for specialists so that they may better diagnose cases and provide effective therapies and proposed antiviral and anti-inflammatory drugs for patients.

## Introduction

Pneumonia with unknown cause has been spreading in Wuhan city (Hubei province, China) since December 2019, while none of the previous vaccines or treatments has been effective. When more than 1,000 patients with coronavirus were identified, the world health organization (WHO) named it coronavirus disease 2019 (COVID-19) on February 2020. WHO also declared a state of emergency before finally recognizing it as a pandemic outbreak on March 11, 2020. Over 7,458,000 infected cases were confirmed in more than 200 countries on June 11, 2020. Major outbreaks of this disease have been reported in China, Italy, South Korea, and Iran, later spreading to many other countries. Mortality of COVID-19 has been approximately 34,114 people in 215 countries, including 4,634 in China, 34,114 in Italy and 8,506 in Iran (1, 2).

Based on the initial reports, the majority of patients were men over 50 years of age. Notably, it was determined that many of them worked in large seafood markets. The reason may be zoonotic transmission between pets and live wild animals traded in the markets; however, scientists observed that the disease is quickly transmitted among the individuals (3, 4).

COVID-19 (also known as SARS-CoV-2) is a RNA virus belonging to the coronaviridae family. It is widely distributed among humans and other mammals. In most cases, the primary symptoms, such as fever, ague, cough and myalgia, fatigue or similar symptoms were observed (5).

Clinical presentations of COVID-19 vary across a broad spectrum of patients with problems ranging from asymptomatic infection and mild upper respiratory tract disease to severe viral pneumonia with respiratory failure and death (6). Morbidity in patients with COVID-19 is secondary to severe alveolar injury and progressive respiratory failure (7). In patients who are mostly between 30 and 79 years old, the symptoms start a few days after virus infection, although can sometimes appear later. In some individuals, there may be no symptom at all (8). WHO has announced that incubation period of the disease lasts up to 14 days, but some researchers believe that it can lasts up to 20 days (9). Based on this, in addition to the highly infectious nature of SAR-CoV-2, the most appropriate approach is quarantine (10).

One of the most successful quarantine applications was in Wenzhou, a city in China that is 900 km away from Wuhan. Wenzhou has a population of 9 million and was one of the first and worst hit cities in of Hubei Province with a more than 504 confirmed cases by the end of February 2020. Wenzhou achieved a remarkable success in controlling the disease outbreak by systemic and rigorous reinforcement of social distancing and home quarantines. The municipal government of the city enforced 25 preventive measures to control the outbreak of coronavirus, such as traffic limitation and reduction of people congregating in both public and private areas, etc. Furthermore, different places were sterilized daily, people used masks and air filtration was implemented when possible. Those who detected positive for COVID-19 (using laboratory tests) and people who returned from the virus epicenter of Wuhan were isolated and carefully monitored either in the hospital or at home. Additionally, only one member of a family could go out to buy home supplies as a method to further contain the spread of the virus. Overall, we know that COVID-19 has caused a great deal of fear and anxiety among people around the world, but this issue have been controlled and Chinese people have managed to control the outbreak correctly (10).

On top of effective control of the disease, Wenzhou also set up a great record for the clinical COVID-19 patients resulting in death rate well below 1%, one of the lowest ratios in China. From January 17, when the first COVID-19 patient was confirmed, researchers and clinical specialists proposed and formulated combined approaches. Several additional diagnostic methods were proposed to screen for SARS-CoV-2, such as computerized tomography Computerized tomography (CT)-scan polymerase chain reaction (PCR), value of C-reactive protein (CRP), immunoglobulin G (IgG) and immunoglobulin M (IgM) levels (11).

There are several additional factors that impact the clinical prognosis of this disease, such as gender, age, regulation of the immune system, physical and nutritional status and expression of human leukocyte antigen (HLA) genes (12). Additionally, some studies showed that HLA expression changes like HLA-A 02:02, HLA-B 15:03, HLA-C 12:03, HLA-B 54:01, HLA-B 07:03, HLA-Cw 08:01, DRB1 03:01, and especially HLA-B 46:01 were significantly associated with highrisk respiratory infectious diseases, like severe acute respiratory syndrome (SARS), middle east respiratory syndrome (MERS) and flu. They can influence the severity of the disease, immune response of the host and susceptibility to these respiratory infectious diseases (13). One study showed that evaluation of HLA classes can be effective in predicting whether a subject will be resistant to infection. It was suggested that HLA-B 46:01 and HLA-B 15:03 are linked to this disease. This study showed that subjects with HLA-B 46:01 are more susceptible to COVID-19. In addition, HLA-B 15:03 potentially plays a role in the release of SARS-CoV-2 peptides in humans. Therefore, this factor could be useful in predicting the most vulnerable individuals to COVID-19 (13). Notably, lymphopenia development, chest CT-scan status, real-time PCR test, rate of oxygen intake, levels of CRP, D-dimer and ferritin levels are all important in determining clinical management (14).

The mortality rate of COVID-19 is about 2% (15), but this is significantly higher in patients with comorbidities and older individuals. In patients over 80 years of age, the mortality rate is 14.8%. Patients with diabetes, cardiovascular disease, chronic respiratory disease, hypertension and cancer are also at increased risk (7.3%, 10.5%, 6.3%, 6% and 5.6%, respectively). Furthermore, the mortality rate is around 3.8% in hospital personnel. These people are already operating in a high risk environment and this higher mortality rates suggest that that disease will be more severe (3).

The outbreak of COVID-19 has lasted several months and there is a lack of definitive information, widely available and accurate diagnostic methods or effective therapy to manage the disease. We also do not have sufficient information about risk factors that may exacerbate the disease and increase mortality in these patients. Interestingly, Wenzhou has enforced multiple precautionary measures to prevent the outbreak of this virus at the right time, so this study suggests that all countries should adopt the proper approaches and standards to inhibit this disease. While this challenging issue can be controlled, scientists and physicians across the world must communicate with each other in order overcome this outbreak. WHO has recommended knowledgetransfer required to increase awareness of the outbreak, diagnostic, therapeutic and pharmacological methods in COVID-19 field. Therefore, Chinese and Iranian clinical and pulmonary specialists shared several questions regarding clinical and molecular diagnostic methods, different drugs applications and proposed therapeutic methods. Chinese scientists then responded to them based on their own experiences in dealing with COVID-19 patients. This article can be used as a reference tool for practitioners and specialists to improve the common treatment and diagnostic methods for SARS-CoV-2 and the related disease, COVID-19 (15). Iranian clinicians prepared some questions about the diagnosis, therapies, types of drugs, etc. These questions are listed in Table 1. The questions were gathered by pulmonary specialists and scientists and were asked during a webinar. Then Chinese specialists answered these questions based on their own experiences.

In general, WHO guidelines recommend that noninvasive ventilation (NIV) can be used for one hour for patients with severe COVID-19, whereas China’s health commission recommends that NIV can be used for maximally two hours in patients with a PaO_2_/FiO_2_ of 150- 200. It is important to use NIV in combination with other therapies. If there is extended use for a longer period of time, such as an entire day, it is important to closely monitor disease progression. We consider it will not be helpful to try NIV for more than one day if there is no improvement in the oxygen saturation levels. In some cases with COVID-19, respiratory rate of patients will not increase even though they have moderate to severe hypoxia (16).

For COVID-19 patients with blood oxygen saturation ≤93% or respiratory rate (RR)≥30 times/min on room air, initial oxygen therapy should be started immediately at 5 l/min and the oxygen should be selected based on the severity of hypoxia as well as the available treatment devices, including nasal catheters, simple masks, oxygen storage masks, etc. If the oxygen storage mask absorbs oxygen at a flow rate of 10-15 l/min and the pulse oxygen saturation is still ≤90% or RR≥30 times/min, then severe acute hypoxic respiratory failure or acute respiratory distress syndrome (ARDS) should be considered and the patient should receive further respiratory support treatment as soon as possible (16).

**Table 1:** The questions which discussed during the webinar and addressed in the paper


No	Questions

1	According to the guidelines, NIV is not applicable for the severe virus infections. However, do you have any experience, which shows its positive effects on COVID-19 patients? What are the exact indications for using NIV for these patients? Shouldn’t we be worried about spreading the virus by using this method?
2	Do you have experience with ECMO for your COVID-19 patients?
3	What are your experiences regarding lung CTs of the COVID-19 patients?
4	Do you have any experience about sampling for PCR diagnosis? Which sampling protocol has more accuracy?
5	How many patients do you have with positive CT which their PCR tests are negative? And, do you categorize these patients (CT+ and PCR-) as the COVID-19 positive/negative patients?
6	Do you have any experience on using BAL for COVID-19? Do you think it works?
7	Do you have any experience about using "Favipiravir" (a new pharmaceutical drug, also named Avigan) for COVID-19 patients? If yes, on how many patients and what was the result?
8	What is the impact of Glucocorticoids for COVID-19 patients? If it is useful, what is the exact steroid type that you use? What is its dose per day and the interval of administration?
9	Do you have any experience about using dexamethasone for patients in ICU?
10	Does plasma exchange work? Do you have any experience on plasma exchange in people who are already cured?


NIV; Non-invasive ventilation, ECMO; Extracorporeal membrane oxygenation, CT; Computerized tomography. PCR; Polymerase chain reaction, ICU; Intensive care unit, BAL; Bronco-alvage, and COVID-19; Coronavirus Disease 2019.

**Fig.1:**
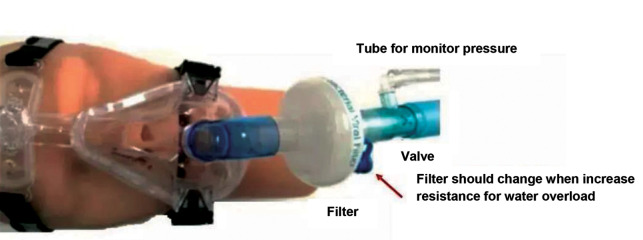
Location of filter near the patient, while non-invasive ventilation (NIV) (between the mask and exhalation valve) is marked by red arrow.

**Fig.2:**
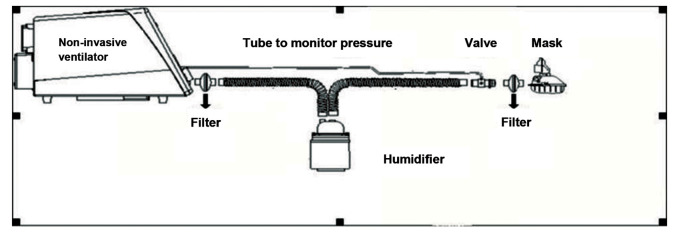
Location of filter, connected in gas outlet of non-invasive ventilation (NIV) and marked by black arrow.

For patients with severe acute hypoxic respiratory failure and mild-to-moderate ARDS (150 mmHg <PaO_2_/FiO_2_≤300 mmHg), high-flow nasal cannula oxygen therapy (HFNO) is preferred, followed by NIV. Changes in the patient's condition should be closely monitored by medical staff who is skilled in performing tracheal intubation during treatment with HFNO or NIV. In patients with mild ARDS who cannot tolerate HFNO, NIV treatment can be attempted. But, it is not recommended to switch therapeutic approach into NIV in patients who have failed HFNO. Regarding the application of NIV, it is important to use the disposable exhalation valve instead of the mask-integrated valve and platform valve. A filter can be added between the mask and the exhalation valve (as shown in Fig.1 and Fig.2). However, this can cause problems, as the filter will increase resistance for water and it can cause water overload. Therefore, filter use should be carefully considered and in case of increasing resistance, it immediately needs to be replaced. The clinically used passive expiratory valve usually includes single orifice valve, mute valve, platform valve and mask-integrated valve. The vent hole of the mask-integrated valve is located on the face of mask, and the exhaled gas is directly discharged. This increases the risk of aerosol transmission of COVID-19 (16, 17).

**Fig.3:**
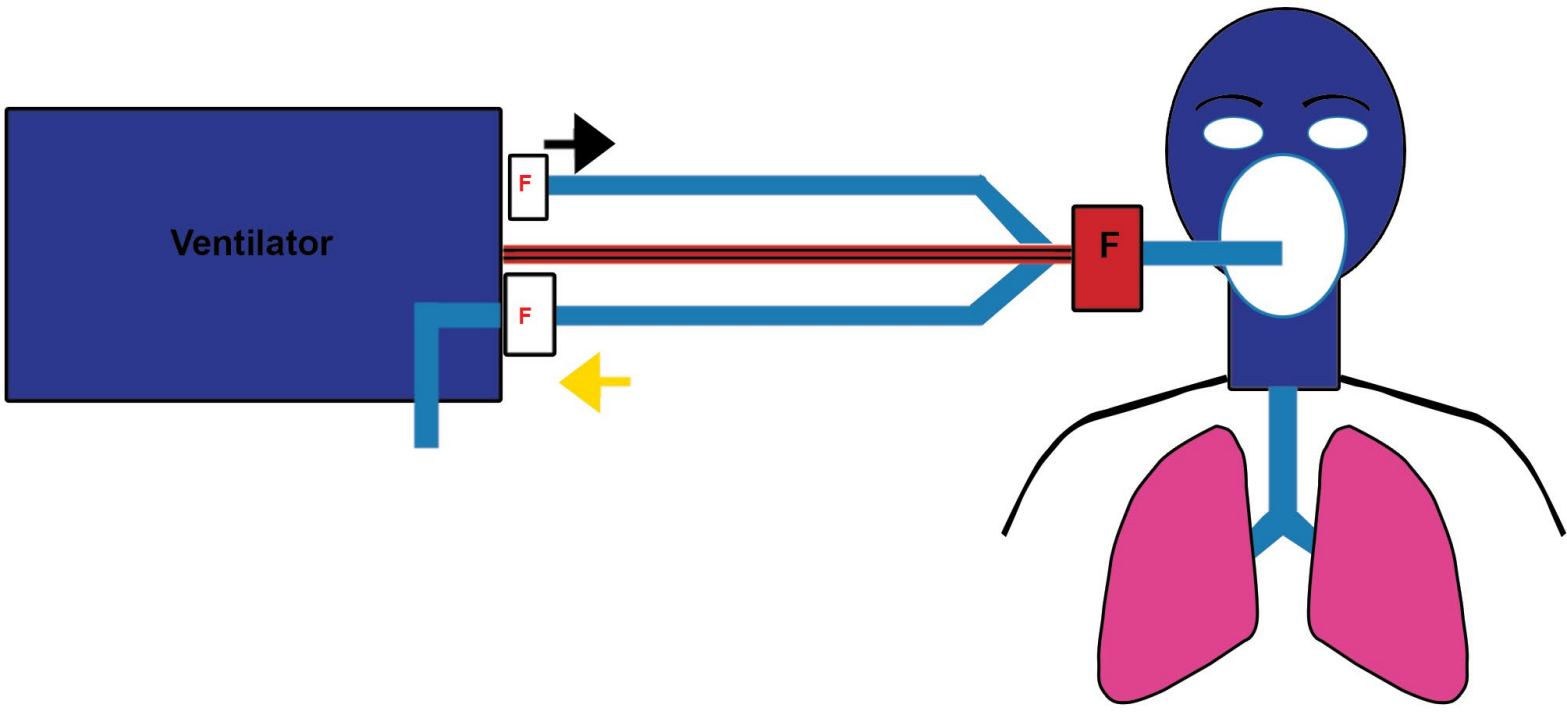
Location of filter (F) in the patient with tracheal intubation filter.

When patients are under tracheal intubation and on ventilator support, it is recommended to use a heat and humidity exchanger (passive humidification) with bacterial filtration function for the humidification of the invasive ventilator (including transfer ventilator). Alternatively, a dual-heating wire humidifier can also be used for active humidification. It is recommended to use a closed-type automatic water renewal humidification tank or a self-made semi-automatic humidification tank as a water-adding device (Fig .3). It should be noted that active humidification is not recommended to increase the filter at the Y-shaped pipe, since this will increase water content of the filter, consequently increasing the respiratory resistance (16).

In China, ECMO is often used in the very late stages of disease and the crude mortality rate is not known. Among our six patients, two patients successfully weaned off ECMO and one passed away, while the other three patients are still under treatment. Veno-venous extracorporeal membrane oxygenation (VV-ECMO) was chosen for five patients and veno-arterial extracorporeal membrane oxygenation (VA-ECMO) was selected for one patient (17). Sometimes, patients with severe COVID-19 deteriorate quickly. If the patientپfs hypoxia does not improve after standard ARDS treatment, ECMO should be started to avoid multiorgan damage caused by either hypoxia or over-ventilation. Based on the previous relevant clinical studies and the recommendations from the International Organization for Extracorporeal Life Support, ECMO should be used in the setting of hypoxic respiratory failure (primary or secondary) caused by any etiology. When the risk of death reaches or exceeds 50%, ECMO should be considered. And by reaching risk of death to 80%, ECMO treatment should be initiated. ECMO can be started under optimal ventilation conditions (FiO_2_≥0.8, tidal volume 6 ml/kg, Positive end-expiratory pressure (PEEP)≥10 cmH_2_O), if there is no contraindication and it meets one of the following conditions: 1. PaO_2_/FiO_2_<50 mmHg More than three hours; 2. PaO_2_/FiO_2_<80 mmHg exceeds six hours; 3. FiO_2_=1.0, PaO_2_/FiO_2_<100 mmHg; 4. arterial blood pH value <7.25 and PaCO_2_>60 mmHg more than six hours and respiratory rate >35 times/min; 5. when the respiratory rate is >35 times/min, the blood pH is <7.2 and the plateau pressure is >30 cmH_2_O and 6. severe air leak syndrome carrying cardiogenic shock or cardiac arrest (17).

The pathophysiology of critical COVID-19 is heterogeneous in lung injury, but its clinical characteristics are different from those of the other ARDS (18). Refractory hypoxemia: ARDS, causing by COVID-19, decreases lung volume due to the collapse of a large number of alveoli, decreased lung compliance and hypoventilation, as well as the blood flow ratio disorders. Of these etiologies, decreased pulmonary ventilation and blood flow ratio disorders are the main causes of hypoxemia. Interstitial pulmonary edema in ARDS compresses the small airways and reduction of surfactants causes partial alveolar collapse, resulting in inadequate ventilation of the corresponding lung unit. This, in turn, leads to a decrease in pulmonary ventilation and blood flow ratio, causing functional shunting with extensive atelectasis and alveolar edema. Local lung units have only limited blood flow without ventilation. That is true shunt and the main cause of refractory hypoxemia (19). Hypercapnia that is difficult to correct with conventional ventilation: COVID-19-induced ARDS has different clinical manifestations from the other ARDS and some patients will develop hypercapnia. Mechanism of hypercapnia is due to the non-uniformity of lung damage caused by the new coronavirus. This results in excessive expansion of normal ventilated lung tissue around the collapsed lung tissue. This leads to increased alveolar dead space, CO_2_ retention and hypercapnia (19). Low lung expandability: ARDS caused by COVID-19 is a typical endogenous ARDS in the lungs, but response to lung expansion and PEEP is poor. The main mechanism is that local lung injury from virus causes high pressure to open the alveoli. Even if the routine lung expansion is attempted, the collapsed alveoli often cannot be expanded, but the surrounding alveoli are over-expanded. This in turn exacerbates the patient’s hypoxemia and hypercapnia. We performed invasive mechanical ventilation on 12 patients with severe COVID-19 and used lung expansion/dilation index to evaluate the results. We found that lung expandability of 11 patients was very low. Therefore, ARDS caused by COVID-19 should be evaluated for lung expandability during further mechanical ventilation treatment to determine the appropriate ventilation strategy (19).

Acute right heart dysfunction: Hypoxemia, hypercapnia and secondary massive alveolar collapse due to COVID-19 will cause acute pulmonary hypertension, followed by acute right-sided heart failure. The associated hemodynamic changes severely impact the patient’s clinical prognosis. The mechanism of pulmonary hypertension, induced by ARDS, starts with alveoli collapse, leading to hypoxia and hypercapnia. This opens calcium ion channels on the cell membrane, depolarizing the cell membrane and increasing the concentration of calcium in the cytoplasm. Ultimately, this leads to vasoconstriction and subsequent pulmonary hypertension. The alveoli collapse might be associated with collapse of alveolar blood vessels, which in turn increases pulmonary arterial pressure. Under inappropriate mechanical ventilation, even if collapsed alveoli are re-expanded, alveolar hyperinflation may still occur in non-gravity-dependent areas of the lung. Alveolar hyperinflation compresses surrounding vascular structures. This increases pulmonary vascular resistance and leads to acute right-sided heart failure (19, 20).

Indications of conventional ARDS prone position ventilation include moderate to severe ARDS [oxygenation index is less than 150 mmHg (1 mmHg=0.133 kPa)]. Additionally, severe hypoxemia and/or hypercapnia should be actively given prone position ventilation. However, severe COVID-19 patients should be treated with prone ventilation as soon as possible. Due to the highly contagious nature of the new coronavirus, medical personnel need to perform tertiary protection. Therefore, the workload of medical personnel is further increased by implementing the prone position. The indication of prone ventilation in COVID-19 patients should be distinguished between severe and critical. For patients with severe disease, active prone ventilation can delay the progression from severe to critical. The prone position should be maintained as long as possible, depending on the tolerance of patient. Prone position ventilation is required for protective lung ventilation in critically ill patients whose hypoxemia and/or hypercapnia have not improved. Prone position ventilation should also be performed in the patients undergoing ECMO therapy. There is medical evidence that ECMO combined with prone position ventilation can improve the clinical prognosis of patients with severe ARDS secondary and COVID-19. It should be noted that COVID-19 patients could have low lung expandability. Therefore, prolonged ventilation should be maintained for at least 12 hours. Additionally, oxygenation changes and respiratory mechanics should be regularly evaluated (21).

One of the most common methods of oxygen therapy is nasal catheter application for infected cases in the mild stage. While this is a non-invasive method, the patient needs to be assisted by others. Therefore, it might not be comfortable for patients. One study confirmed that high-flow nasal cannula (HFNC) is a proper oxygen therapy method for severe and critical stages. HFNC has appropriate outcomes including exposure of warm and humid gas through patients’ nasopharynx, which leads to a decrease in metabolic reactions in body, reduction of intubation, improving clinical condition of infected cases with acute respiratory failure; as well, physicians can control and apply this method easily. Despite these positive outcomes, HFNC is not an appropriate option for cases with ARDS in severe stage; the gas flow is high, so the possibility of bio-aerosol dispersion increases (22).

CT-scan and PCR have been used to detect infected cases and viral particles; however, it is important to evaluate sensitivity and specificity of these methods, as well as to assess other practical techniques.

CT scans are very important tools in COVID-19 diagnosis and forming a differential diagnosis, but they are also useful for determining patient prognosis. CT results in COVID-19 cases generally show patchy ground-glass opacities involving multiple lobes. These can be with or without consolidation and are mainly in the peripheral zone. There may also be associated reversed halo signs, vascular thickening, a crazy-paving pattern, or an air bronchogram sign. Patients who have CT scans showing multiple lesions need to be carefully monitored. Despite appearing well on initial presentation, these patients tend to have poorer prognoses and can potentially deteriorate very quickly to severe or critical conditions. Several specialists believe that CT-scan is one of the most useful and widely available medical tools for obtaining a better diagnosis. Clinicians should use the results of the CT scan in combination with reverse transcription polymerase chain reaction (RT-PCR) testing to determine the best course of action. The analysis of advantages of CT-scan images showed that this method is highly sensitive (14).

In China, there is a nationally standardized RT-PCR kit, but its sensitivity can sometimes be as low as 50 percent. The sensitivity mostly depends on the samples (nasal pharyngeal swab shows more positive cases than oral pharyngeal swab; broncho alveolar lavage fluid (BALF) is the most accurate one, sputum is less sensitive than BALF but it may be better than swab). IgM and IgG measurements also are performed which are really helpful. In cases where the results of PCR and CT-scan are different (i.e. CT manifest as typical virus pneumonia and PCR is negative), it must be considered that some errors might have occurred in the PCR results due to the quality of the sample; the process of laboratory testing or the testing kit itself (14). However, these issues tend to be very rare recently because the testing is defined as a combination of tests including the IgG and IgM measurements (11). RT- PCR and the technology of high-throughput sequencing have been used to detect SARS-CoV-2, but high-throughput sequencing is not common because the instrument itself is expensive. Therefore RT-PCR has been used and its sensitivity and specificity have been confirmed (14).

In Wuhan, a physician tried to use bronco-alveolar lavage (BAL) for critical patients who receive ECMO, and it was reported that after 2-3 trials of BAL and removal of some plasma liquid, the lungs became clear, but the final results of the treatment are not clear (When BAL was performed, the patient needed to be completely sedated and was administered a muscle relaxation drug. The physician needed to protect himself with positive pressure head cover) (17, 23).

Furthermore, several additional methods and clinical symptoms have bee also used to detect COVID-19 patients. These methods are explained in following and summarized in Table 2.

Learned from autopsy, there is a lot of viscous mucus in the airway and alveoli with obstruction of the airway in the patients. In these cases, acetyl cysteine and bronchodilator nebulization and tablets of acetyl cysteine are necessary (20).

The other unique feature in critically COVID-19 compared to influenza is that in COVID-19 more patients tend to have a gastric tension (belly tension) (24).

The low oxygen level is the most distinctive feature of COVID-19. In most of other situations, hypoxia is usually associated with tachycardia. However, in part of COVID-19 patients, despite hypoxia, heart rates may appear in normal range (silent hypoxemia) (17, 25).

In some cases, the patient’s bilirubin level elevates, particularly when they are given antiviral drugs that have hepatotoxic effects. One study report liver enzymes [Aspartate aminotransferase(AST) and Alanine transaminase (ALT)] were unusually increased in some infected cases with COVID-19 and the rate and extent of ALT and AST elevation in severe COVID-19 patients were higher than those in nonsevere patients (11, 23). In normal liver tissues, it is observed that ACE2 was just expressed in epithelial cells of bile duct; however, its level is so low in hepatocytes (7, 11).

Markers consist of CRP, procalcitonin, ferritin, D-dimer, lymphocytes, interleukin 4 (IL-4), interleukin 6 (IL-6), interleukin 10 (IL-10), tumor necrosis factoralpha (TNF-a), interferon gamma (INF-y) which can help evaluate clinical progress, alert severe and critical tendencies and provide a basis for the formulation of treatment strategies. When patients have high temperature, high CRP and IL-6, high ferritin, increase D-dimer, progress infiltration in CT scan, deteriorate of oxygen index, that means the patient may shift to critically ill disease (11).

Other diagnostic methods have been proposed, including IgM/IgG and enzyme-linked immunosorbent assay (ELISA) kits for coronavirus, this assay could aid to detect virus particles in suspected cases (11, 14). One research study showed that these antibodies are produced against the N protein of SARS-CoV-2. It is also observed that the specificity of IgM and IgG is 100% and 95% respectively and the sensitivity of IgM and IgG is 77.3% and 83.3%, respectively (26). In addition, blood culture and detection of nucleic acids are other ways to diagnose SARS-CoV-2. However, the technique of nucleic acid detection is based on whole genome sequencing and while it is accurate, this method is expensive (14).

**Table 2:** Further clinical/ laboratorial diagnostics for COVID-19 (more details in B-4)


Indicators	Diagnostic Method	Reference

**Clinical**		
Obstructive airway	Observational	(20)
Gastric tension	Observational	(24)
Hypoxia	Observational, Oxygen level measurement	(17)
Silent hypoxia	Observational, Oxygen level measurement	(17, 25)
**Laboratorial Indicators**		
AST, ALT, Bilirubin, ACE2	Radioimmunoassay, Spectophometer	(7, 11)
CRP, Procalcitonin, Ferritin, D -dimer, Lymphocytes, IL-4, IL-6, IL-10, TNF-a, INF-y	ELISA, Radioimmunoassay	(11)
IgM/IgG levels	Serological test, ELISA	(11)
Detection of nucleic acid	Whole genome sequencing	(11)
Virus detection	Blood Culture	(11)


AST; Aspartate aminotransferase, ALT; Alanine transaminase, ACE2; Angiotensin-converting enzyme 2, IL-4; Interleukin 4, IL-6; Interleukin 6, IL-10; Interleukin 10, TNF-a; Tumor necrosis factor-alpha, INF-y; Interferon gamma, and ELISA; Enzyme-linked immunosorbent assay. IgM; Immunoglobulin M, and IgG; Immunoglobulin G.

Here, Chinese and Iranian clinicians discussed about some drugs and therapies in detail and Chinese clinicians expressed their experiences in treating various conditions. To better understand the latest situation on this topic, more recent clinical investigations on new drug or drug combinations has been disscussed separately.

Favipiravir, which is also named Avigen, has been used to treat COVID-19 patients. Chinese specialists do not use Favipiravir at their center, as the primary indication for this drug is influenza. However, a recent clinical trial using Favipiravir to treat COVID-19 in China has shown encouraging results. Below is a brief summary of the study. (This report translated from the Biotechnology Network from Chinese). Results of a "clinical study on the safety and effectiveness of favipiravir in the treatment of patients with new coronavirus pneumonia (COVID-19)" (Registration Number: ChiCTR2000029600). Research suggests that Favipiravir may improve the clinical course of this new coronavirus pneumonia by accelerating viral clearance. This research was completed by the National Engineering Research Center for Emergency Prevention and Control Engineering and the Third People’s Hospital of Shenzhen City. Viral clearance is the main internationally accepted gold standard for evaluating clinical efficacy of antiviral drugs. In this clinical study, 35 patients with common new-type coronavirus pneumonia who met the eligibility criteria were treated with Favipiravir (3,200 mg on the first day, 1,200 mg/d on the 2^nd^ to 14^th^ days, divided into two oral doses, the course of treatment until the virus was eliminated, up to 14 days). The study also included 45 patients with COVID-19 who were treated with Lopinavir/Ritonavir tablets (400 mg/100 mg, twice daily, orally) matching for age, gender, and disease severity to the control group. The median time from drug administration to viral clearance, the rate of improvement of chest imaging on day 14 of treatment, and safety were compared between the two groups. The results showed that all baseline characteristics of the two groups of patients were comparable. The median time to viral clearance was shorter in the Favipiravir treatment group, with a median (interquartile range) of 4 days (2.5-9 days) and a control group of 11 days (8-13 days), with significant differences between the two groups (P<0.001). After controlling for potential confounding factors (age, time to onset of symptoms, fever etc.), Favipiravir remains an independent influencing factor for improved chest imaging and early viral clearance. Compared with the control group, the Favipiravir group had fewer adverse reactions and was better tolerated (27).

In general, the consensus reached by experts is that glucocorticoids are not routinely used, and the indications should be strictly controlled. The general agreement is that glucocorticoids are not required in light and common type patients [In China, COVID-19 patients can be divided into four types: mild (only RTPCR positive without pneumonia); moderate, also called as common (RT-PCR positive with pneumonia in CT); severe, and critical pneumonia).] and should only considered in severe and critically ill patients. The indications are established by different criteria including progressive deterioration in oxygenation indicators, quick progression of disease on imaging, and excessive activation of the body’s inflammatory response. Additionally, glucocorticoids should be considered if patients have any of following 4 conditions in conjunction with COVID-19: 1. confirmed new COVID-19 pneumonia within the past 10 days that demonstrates rapid progression on imaging; 2. severe hypoxemia (respiratory failure); 3. common type patients with acute exacerbations (high fever, dyspnea) and severe and critically ill patients; or 4. the deterioration of oxygenation index, with or without rapid progress in imaging. To summarize, most experts do not rule out the use of glucocorticoids, but they should be applied early in the critical illness (usually within 10 days of the disease course). Furthermore, mild and moderate patients usually do not need to use glucocorticoids. They should be administered to severe and critically ill patients in the early stages have one of the following three criteria: 1. progressive deterioration of oxygenation index, 2. rapid progression of lesions on imaging and 3. excessive activation of the body’s inflammation (28).

**Fig.4:**
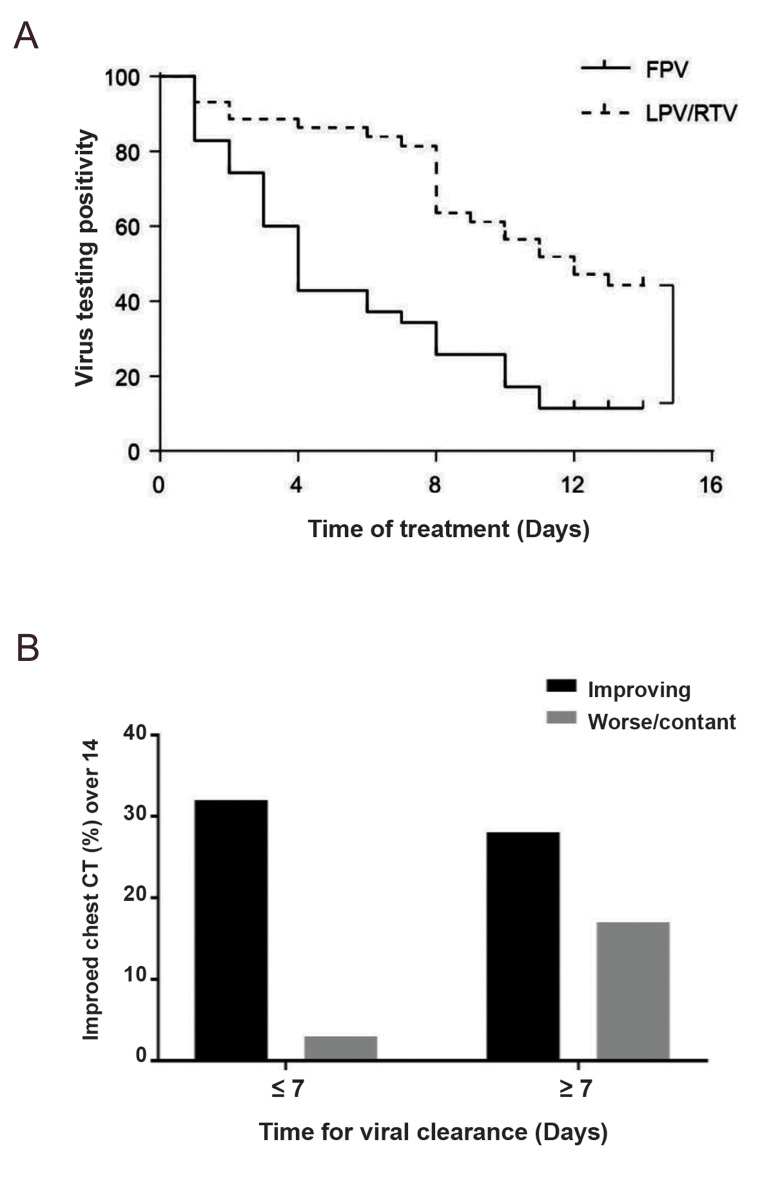
Favipiravir effects on virus testing positivity and chest CT. **A.** Time for virus clearance and survival curve.** B.** Influence of virus clearance rate on the chest CT at day 14 after treatment. CT; Computerized tomography, FPV; Favipiravir, and LPV/RTV; Lopinavir/ritonavir

In theory, glucocorticoids are best used during periods when viral replication is inhibited (whether caused by an antiviral drug or enhanced immune response) and the body’s inflammatory response is very intense. It is difficult to grasp the state of excessive activation (inflammatory storm) of the body’s inflammatory response (general situation: about 10 days into the course of the disease). Here is a personal opinion for clinical reference; the new coronavirus infection meets the severe clinical manifestations and can be considered as an "inflammatory storm" state when the following conditions are met: persistent high fevers, progressive deterioration of oxygenation, rapid progression of lesions on imaging, continuous decline in the absolute value of lymphocytes, and a significant increase in interleukin 6. The dose and duration of glucocorticoids (Aprenosaurus as a representative; this is the same as methylprednisolone of China): as a representative]: the dose is 40~160 mg/d, for no more than 7~10 days. Glucocorticoids are mainly effective in patients who have developed lung injury. In general, the principle of application is an individualized treatment: it should be administrated at the right time (early, usually within 10 days of the disease), to the appropriate patients (severe and critically ill patients who experience excessive inflammation in the body), with an appropriate dose (usually a medium dose of 40~160 mg/d, and large dose shock is not recommended) and a short course of treatment (not more than 7~10 days). As well, paying attention to the adverse effects of glucocorticoids is crucial. Analysis of contraindications before using glucocorticoids: Glucocorticoids should be used with caution in the following cases: diabetes, known allergies to other steroids, high blood pressure, epilepsy or delirium, glaucoma, active gastrointestinal bleeding in the last three months, problems with hypokalemia correction, secondary microbial infections, immunosuppressed condition (like chemoradiation, major surgery, acquired immune deficiency syndrome) and severe lymphopenia (absolute value <300 / μl).

Nowadays, the use of dexamethasone in ICU patients is a difficult decision to make for clinicians, as the general understanding is that dexamethasone has long lasting effects. Furthermore, in practice, physicians are generally more used shorter half-life methylprednisolone. Investigating the Clinical-Trials. gov website showed that 291 projects have been conducted on COVID-19; Among these projects, 109 studies referred to pharmacological and therapeutic objectives for COVID-19 of which 87 of these trials are in different phases including 4, 36, 36, and 11 studies in phase I, II, III, and IV respectively. On the grounds that COVID-19 is closely related to SARS and MERS, it has been recommended that the drugs which are used to treat SARS and MERS, might be appropriate for COVID-19 treatment too. Therefore, a list of pharmacological agents has been proposed to be investigated. All of the proposed drugs should be precisely evaluated in terms of adult dose, contraindications, toxicities, major drug-drug interactions, etc. Recently, clinicians prescribe Nintendanib and Perfenidone to patients with COVID-19 and idiopathic pulmonary fibrosis. These drugs have anti-oxidant and anti-inflammatory activities. Chloroquine/Hydroxychloroquine which is known as anti-malaria drug, showed anti-inflammatory activities when administered for COVID-19 patients, although its efficacy remains hotly debated. It also is able to inhibit viral entry to host cells by different mechanisms, such as inhibition of proteolytic process. Lopinavir/ Ritonavir is practical for acquired immunodeficiency syndrome (AIDS) treatment and also inhibits 3-chymotrypsin-like protease in SARS. Therefore, the drugs can be proposed for therapeutic purposes in COVID-19; however, some observations showed that these caused side-effects like hepatoxicity and are not appropriate for all conditions of the disease. Ribavirin which inhibits viral RNA-dependent RNA polymerase and has antiviral activity against nCOVs, has been used against SARS and MERS. However, several side-effects such as hematologic and liver toxicity were reported. High dosage of this drug should be used to inhibit viral replication which may cause adverse outcomes in patients. Due to its side effects, its application has been limited in COVID-19 treatment. Umifenovir (Arbidol), which has been confirmed to treat influenza in China and Russia, targets interaction of S protein/ACE2 and inhibits formation of the components of the viral envelop. A randomized control trial has been launched recently in China to investigate its effects on COVID-19. Another drug with destructive effects against RNA viruses is Remdesivir. This drug is an appropriate agent to prevent lung hemorrhage and decreases viral lung titers and used against Ebola virus and now suggested for patients with COVID-19. (28).

Several studies showed that Immunoglobins are applicable in several viral diseases such as Ebola, influenza and HIV-1. Therefore, immunoglobins have been considered as a potential therapeutic agents in COVID-19. In addition, it was reported that corticosteroids such as methylprednisolone, hydrocortisone and dexamethasone are effective in SARS-CoV. Nowadays, methylprednisolone is prescribed with oseltamivir, antibiotics and oxygen therapy in the patients infected COVID-19. Other agents which were used in SARS, MERS, or AIDS and are proposed for COVID-19 included Darunavir, recombinant human interferon α2β and thalidomide (28).

There are two criteria for performing plasma exchange: critical severe COVID-19 and COVID-19 that is complicated by liver damage and cytokine storm (high levels of interleukin 6). However, plasma exchange is not a routine procedure and is certainly not a widespread practice in the Chinese clinical community. Some treatments are usually applied, including oxygen therapy and application of a wide range of antibiotics, to prevent secondary bacterial infections (28, 29).

Researchers believe that blockers of RNA-polymerase can be effective in reducing RNA-virus proliferation in the body. In addition, some studies recommended inhibitors of Chymotrypsin-like (cinansrin and flavonoids) and papain-like protease (diarylheptanods), also use of blockers to prevent binding S-protein with ACE2, may be effective strategies to decrease this infection.

Other studies demonstrated that types of vaccines, monoclonal antibody, and plasma substitution could be appropriate for the treatment of COVID-19. Hemoperfusion and plasma exchange are applicable for COVID-19 and they cause removal of plasma proteins, cytokines, and toxic factors. However, it is observed that hemoperfusion has adverse outcomes such as reduction of blood glucose, calcium, neutrophils, etc. Furthermore, plasma exchange leads to a decrease in coagulation proteins and antibodies. Therefore, coupled plasma filtration adsorption is proposed. This technique uses a combination of hemoperfusion and plasma exchange and it removes cytokines in infected cases with COVID-19. Another proposed treatment for COVID-19 includes mesenchymal stromal cell (MSC) transplantation. These cells, which include several subtypes, are able to modulate the immune system and inflammatory reactions. Based on pathological characteristics of infected patients, this therapy can be proposed, especially for severe and critical stages. There are several trials which show this therapy is safe rather than others for patients (28).

In the context of the viral pandemic, one of the most important approaches that researchers and physicians should follow is to communicate effectively and quickly with each other and to establish ways to exchange scientific information about different aspects of diagnostics and therapeutics. This communication happened from the earliest moments of the COVID-19 pandemic between a group of Chinese and Iranian clinicians and medical researchers. Present article is the result of this exchange of views which mainly consists of three categories: A. pulmonary techniques for COVID-19 patient, B. clinical and molecular diagnostic methods for COVID-19 and C) Drug combinations and new therapies for COVID-19. A wide range of antiviral and anti-inflammatory drugs, respiratory therapies, plasma exchange, and application of mesenchymal stromal cells as well as the most common/ novel diagnostic methods have been discussed throughout these three sections and the latest investigations in regards to these have been mentioned where relevant.
